# 

**Published:** 1915-03-13

**Authors:** 


At a meeting of directors of the Glasgow Royal Maternity and Women's Hospital held on Monday afternoon, Dr. Jessie G. McLaren was appointed indoor house surgeon for six months, and Dr. Grace B. Whish outdoor house surgeon for three months from April 3 next.

### Buildings Contemplated.

Bexhill.—The Local Government Board are holding an inquiry into an application by the Bexhill Town Council for sanction to borrow £6,500 for the erection of new isolation hospital buildings at Clinch Green.

Blackadon.—The Plymouth Town Council have approved the extension of the borough mental hospital at Blackadon to accommodate 440 additional patients, and Messrs. Thorneley, Rooke, and Barron, architects, have been instructed to prepare plans. The cost of the extension is estimated at £100,000.

Clydebank.—The Town Council are making inquiries in regard to a site for a tuberculosis hospital.

Dumfries.—The directors of the Dumfries and Galloway Royal Infirmary have agreed to carry out a scheme of reconstruction at the infirmary, at an estimated cost of £2,360.

Gartloch.—At a meeting of the Glasgow District Board of Control it was decided to obtain competitive plans for additional buildings to accommodate 300 patients at Gartlock Mental Hospital, with attendants' houses.

Huddersfield.—The Corporation have decided to build a tuberculosis hospital at Bradley Gate.

Slaley.—The Northumberland County Council propose to build a sanatorium at Slaley, Hexham.

Stockton.—The Guardians have decided to proceed with the preparation of plans for a new infirmary.

Sutton Ford.—The Rochford R.D.C. have adopted plans, submitted by the Joint Hospital Committee, for the enlargement of the Sutton Ford Isolation Hospital. The estimated cost is £2,000. The plans will be sent to the Shoeburyness Urban District Council for their consideration.

### Personalia.

Mr. George Sefton Miller, third assistant medical officer at Lambeth Infirmary, has resigned his appointment in order to join the Royal Army Medical Corps. The Guardians have decided to leave to Dr. Baly, the medical officer, to take such steps as may be necessary to secure the services of a successor.

### NEW PHARMACIST AT KENSINGTON INFIRMARY.

Mr. J. Henry Heworth has been appointed by the Kensington Board of Guardians to be dispenser at the Infirmary, Marloes Road, Kensington, London, in succession to Mr. C. H. Russell, who now retires after about twenty-eight years' service under the Board. The appointment is, in the first instance, for a period of one year, after which time the Guardians will consider the question of making it permanent. Mr. Heworth is known to many institutional pharmacists in London and the East Coast district, for whom he has acted as holiday "locum" for several years. He is a registered pharmacist, and also a member of the Pharmaceutical Society of Great Britain.

### Obituary.

Dr. John Gilliott Garbutt, who was demonstrator of anatomy to St. Mary's Hospital medical school, has died at his residence at 203 Blackfriars Road, S.E. He had reached the ripe old age of seventy-three years, into which he had crowded much good and useful work. Born in Derbyshire, in 1868 he was awarded a B.A. (London) degree with honours, and two years later he proceeded to the degree of M.A. He was a member of the Royal College of Surgeons (Eng.), and studied at St. Mary's. His life was afterwards spent in the borough of Southwark, where amongst the poor he was greatly esteemed and respected. For many years he was medical officer for the London Cab Drivers' Union. Dr. Garbutt had two hobbies, which he cultivated in Southwark not amidst the most congenial surroundings. He was a botanist and an astrologer, and on the roof of his house he was able to indulge his fancies without fear of being disturbed by the street arabs who for many years infested the district. As a musician, he frequently discharged the duties of organist at local churches, being first called upon when only eight years old to perform these duties at a church in the town where he was then staying. He was a Greek scholar of ability. Crowds of the Southwark poor were present at his funeral, and openly manifested their signs of grief at the loss of an old friend.

The sixty-sixth annual report of the Prudential Assurance Company, Limited, states that in respect of the Ordinary Branch the number of policies issued during the year was 65,751, assuring the sum of £6,318,843, and producing a new annual premium income of £424,353. The premiums received during the year were £5,035,625, being an increase of £115,107 over the year 1913. In addition, £10,315 was received in premiums under the Sickness Insurance Tables. The claims of the year amounted to £4,014,658. The number of deaths was 9,351. The number of endowment assurances matured was 24,966, the premium income of which was £136,735. The number of policies in force at the end of the year was 922,505. Industrial Branch.—The premiums received during the year were £8,176,202, being an increase of £301,746. The claims of the year amounted to £3,373,850, including £398,360 bonus additions. The number of claims and surrenders, including 6,731 endowment assurances matured, was 392,883. The number of free policies granted during the year to those policyholders of five years' standing and upwards who desired to discontinue their payments was 103,514, the number in force being 1,947,556. The number of free policies which became claims during the year was 46,364. The total number of policies in force in this branch at the end of the year was 20,085,010 : their average duration exceeds thirteen years. The assets of the company, in both branches, as shown in the balance sheet, are £91,202,344, being an increase of £4,209,341 over those of 1913.

### THE BRITISH FARMERS' HOSPITAL FOR SERBIANS.

The British Farmers' Red Cross Fund informs us that it is equipping No. 4 Serbian unit, to be called the British Farmers' Hospital for Serbians. This is being undertaken in conjunction with the Serbian Relief Fund by arrangement with the British Red Cross Society. £3,000 on account of the above hospital has already been paid to the British Red Cross Society. Another £2,000 to complete this unit will be sent at an early date. The hospital will probably be a tent hospital to facilitate its mobility. £1,000 has also been cabled for use in connection with Dr. Berry's Serbian hospital.

Rates of Subscription: Twelve months, 6/6; Six months, 4/-; Three months, 2/-, post free to any address in the United Kingdom. Abroad, Twelve months, 8/8; Six months, 5/-; Three months, 2/6, post free.—Address The Subscription Dept., "The Hospital," The Hospital Building, 28/29 Southampton Street, Strand, London, W.C.

